# Malignant Disease of the Pylorus

**Published:** 1902-11-29

**Authors:** 


### MALIGNANT DISEASE OF THE PYLORUS.

In seeking a diagnosis, the natural tendency is to place on one side improbable solutions, and it may perhaps be said that one great advantage of experience lies in the fact that the man who has seen many cases is less apt than the practitioner whose work has lain in a more restricted circle to discard improbable explanations as "out of the question." A case of malignant disease of the pylorus in a youth aged 19, is reported by Mr. G. Paul Anning, in the *Lancet*, November 22nd, which well illustrates this point, for according to the statistics given by W. F. Welch, in Hemmeter's work on "Diseases of the Stomach" out of 2,038 of gastric cancer only two occurred between the first and second decades of life, so that the probability of a case at that time of life being cancerous is but small. The patient in question when he came under observation had been ill for eight months, the symptoms being those of a chronic gastritis—pain in the epigastrium, the left hypochondrium, and below the left shoulder-blade, associated with anorexia, lassitude, loss of weight, and subsequent vomiting. Pain was always brought on or aggravated by food, and usually continued for three or four hours or more. There was no hæmatemesis. On examination it was obvious that he had within his abdomen some hard masses, which were situated above the level of the umbilicus in the epigastric region. They were small, lay near to one another, and moved up and down during respiration. The stomach was dilated, but the pylorus could not be felt. The lumps which were felt were regarded as enlarged coronary glands. The vomit showed (by the Congo-red test) the presence of free hydrochloric acid. On consultation with Mr. Littlewood it was agreed that the diagnosis lay between three things—(1) chronic gastric ulcer with pyloric stenosis associated with simple inflammatory enlargement of glands, (2) tuberculous glands, (3) malignant disease. On Mr. Littlewood opening the abdomen the pylorus was found anchored under the left lobe of the liver and enlarged by cancerous growth to a size larger than a child's fist. The gastro-hepatic omentum was studded with large indurated glands, while the coronary glands and those of the great omentum were also much enlarged. Mr. Littlewood performed a gastro-enterostomy which gave immense relief to all the more urgent symptoms. The cancerous process, however, advanced in many directions, and the patient died between eight and nine months after the operation. It has been suggested by Osler that where, at the cancer age, a chronic gastritis exists in association with wasting, which does not tend towards recovery by rest in bed and judicious treatment, at the end of three weeks laparotomy should always be done on the supposition that gastric cancer may be present. But how about cases not at the cancer age? As Mr. Anning justly points out, in the adolescent who insidiously becomes the victim of gastric cancer, there is a tendency to let it drift till at least a palpable tumour can be felt before even the qnestion of cancer is entertained. Let us always be on the *qui vive* for the improbable.

